# Biomaterials for bone regeneration: an orthopedic and dentistry overview

**DOI:** 10.1590/1414-431X2021e11055

**Published:** 2021-06-14

**Authors:** J. Girón, E. Kerstner, T. Medeiros, L. Oliveira, G.M. Machado, C.F. Malfatti, P. Pranke

**Affiliations:** 1 Laboratório de Hematologia e Células Tronco, Faculdade de Farmácia, Universidade Federal do Rio Grande do Sul, Porto Alegre, RS, Brasil; 2 Programa de Pós-graduação em Fisiologia, Universidade Federal do Rio Grande do Sul, Porto Alegre, RS, Brasil; 3 Programa de Pós-graduação em Engenharia de Minas, Metalúrgica e de Materiais, Universidade Federal do Rio Grande do Sul, Porto Alegre, RS, Brasil; 4 Programa de Gradução em Odontologia, Universidade Luterana do Brasil, Canoas, RS, Brasil; 5 Instituto de Pesquisa com Células Tronco, Porto Alegre, RS, Brasil

**Keywords:** Bone regeneration, Tissue scaffolds, Biomaterials, Dentistry, Orthopedics, Metals

## Abstract

Because bone-associated diseases are increasing, a variety of tissue engineering approaches with bone regeneration purposes have been proposed over the last years. Bone tissue provides a number of important physiological and structural functions in the human body, being essential for hematopoietic maintenance and for providing support and protection of vital organs. Therefore, efforts to develop the ideal scaffold which is able to guide the bone regeneration processes is a relevant target for tissue engineering researchers. Several techniques have been used for scaffolding approaches, such as diverse types of biomaterials. On the other hand, metallic biomaterials are widely used as support devices in dentistry and orthopedics, constituting an important complement for the scaffolds. Hence, the aim of this review is to provide an overview of the degradable biomaterials and metal biomaterials proposed for bone regeneration in the orthopedic and dentistry fields in the last years.

## Introduction

Tissue engineering is proving to be a promising field. It consists of the association of cells, biomaterials, and bioactive factors in order to mimic the native tissue, aiming to restore, maintain, or improve tissue function. Bone tissue engineering aims to develop three-dimensional scaffolds to provide the necessary structural support for the formation of a new bone structure, where usually the addition of growth factors and cells contributes to the acceleration of the osteogenic lineage induction.

Bone tissue, when intact, performs critical functions for the human body. It is directly related to locomotion because of the mechanical support that it provides for the body. In addition, bone is responsible for maintaining mineral homeostasis and because of its rigidity, it is the foremost protective barrier of vital organs. As a result of being an active multifunctional tissue, bones are susceptible to injury, which could compromise their function.

Orthopedic and dental bone defects are common problems that can occur due to trauma, infections, neoplasms, congenital conditions, or simply by aging. Therefore, grafts are necessary to replace injured tissue, ensuring a close connection between the implant and the host bone. The materials used in this process must provide an ideal structural environment for cells that participate in the bone healing process.

Autogenous bone is considered the “gold standard” for bone regeneration due to its osteogenic, osteoconductive, and osteoinductive properties. However, its use depends on bone availability, with disadvantages such as the risk of vascular-nervous lesions and morbidity in the recipient bed, thereby limiting its use (1). A less invasive alternative are xenogenic, allogeneic, and alloplastic bone grafts.

The restrictions of xenogenous grafts are their limited capacity to be fully incorporated into the native bone, being present in the implanted area for long periods of time, in addition to the risk of rejection or disease transmission. Allogeneic bone exhibits reduced osteoinductive properties and such grafts have a risk of immunoreactions and transmission of infections (2).

Alloplastic biomaterials have been widely studied in tissue engineering including ceramics, polymers, and metals and these materials can be associated with growth factors or cells. This type of graft has been investigated due to its advantages such as easy handling, great availability of shapes and sizes, and a high variety of resources for its production. In addition, the use of alloplastic biomaterials contributes to the reduction of surgical morbidity, absence of antigenicity, and risk of disease transmission (3).

**Figure 1:**
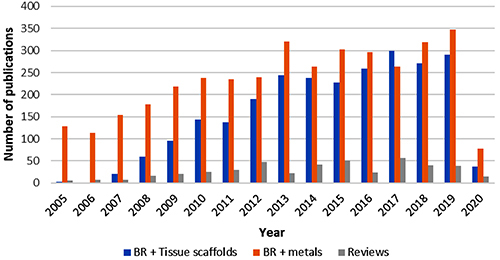
The number of annual publications in the PubMed database from 2005 to May 2020 using the terms: (Bone Regeneration[MeSH Terms]), (Tissue Scaffolds[MeSH Terms]) and (Metals). November 16, 2020. BR: bone regeneration.

Research studies involving the development of scaffolds for bone regeneration have increased considerably over the last fifteen years, and since 2013, the number of publications has remained between 200 and 299 per year, as can be seen in Figure 1. The number of publications related to metals and bone regeneration has also been high over the years, but since 2014, the number of publications involving scaffolds is comparable to that of metals.

Currently, there are several techniques in tissue engineering used to develop scaffolds with different purposes. Some of these techniques are solvent casting, particulate leaching, freeze-drying, gas foaming, powder-forming, sol-gel technique, electrospinning, and 3D printing. For metal biomaterials, the efforts are focused on surface treatment to promote osseointegration.

The aim of this review was to provide an overview of the biomaterials proposed for bone regeneration in the orthopedic and dentistry fields in the last years. This review focuses mainly on degradable biomaterials, without disregarding the important role of metals in orthopedics and dentistry.

## Degradable biomaterials for bone regeneration in orthopedics

Orthopedic regenerative medicine aims to design bone scaffolds and implants able to replicate the biomechanical properties of the host bone. For bone regeneration, a scaffold should be biodegradable and biocompatible, as well as having osteoconductive, osteoinductive, and osteogenic properties (4). In addition, it is desirable that it provides an appropriate exchange of nutrients, promotes vascularization and bone ingrowth, and has a pore size ranging from 100 to 500 µm (5).

### Manufacturing techniques for bone scaffolds

**Figure 2:**
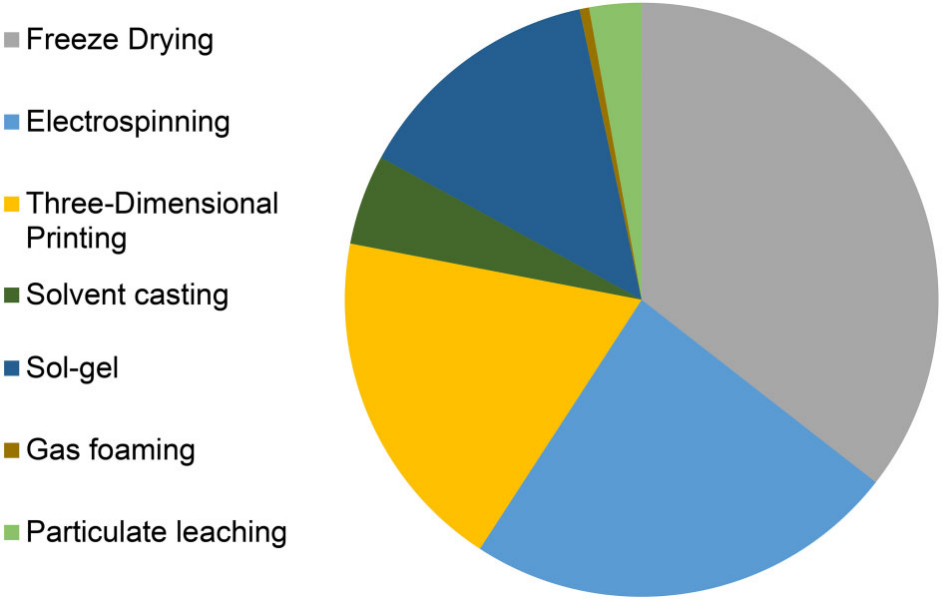
Distribution of manufacturing techniques cited for scaffolds with a bone regeneration objective. Each term has been associated with AND “Bone Regeneration”[MeSH]. June 1, 2020.

Figure 2 shows that in the last 10 years the most cited technique for the development of bone tissue regeneration scaffolds has been freeze-drying, followed by electrospinning, three-dimensional printing, and particulate leaching.

Three-dimensional printing, also known as additive manufacturing, is a technology that has been widely used in bone tissue engineering. It is a fast and strict process that consists of depositing materials layer by layer to produce 3D objects (6). The printed porous scaffold can have controlled parameters, being able to increase the reproducibility of the structure.

Recently, the development of three-dimensional scaffolds based on laser-cutting manufacturing process has been proposed (7). This technique consists of the generation of sheet-based scaffolds in which single sheets are manufactured and cut by a high precision laser that uses templates generated by computer-aided design (CAD). The sheets are then stacked to form a 3D scaffold. The advantages of this technique are that single sheets can be seeded with cells before being stacked, allowing for a spatially uniform cell distribution, as an appropriate cell distribution is difficult to obtain with solid scaffolds.

Using the solvent casting-particulate leaching method, highly porous scaffolds with good interconnections between each pore can be formed, but it can only be used to produce thin membranes of 3-4 mm. This process consists of the mixture of a polymer solution and salt crystals with specific dimensions, followed by evaporation of the organic solvent. The remaining salt particles with the polymer matrix are then leached out by immersion in water, which dissolves the particles and produces a porous structure (8).

Freeze-drying is another technique used to manufacture high porosity polymeric scaffolds in which the polymer solution is frozen, leading to the solidification of the organic solvent that will be later removed by sublimation. Concentration of the polymer solution and the freezing temperature can influence the distribution of the pores and the pore size, enabling the creation of a wide variety of scaffolds with different pore structures (9). As a result, a 3D structure with interconnected pores is developed, although it has low mechanical stability and requires the use of toxic solvents.

Fibrous scaffolds provide a similar architecture to that of the extracellular matrix. Electrospinning is a method that creates an electrically charged jet of a polymer solution through a high voltage system, producing fibers with a thin diameter and a large surface area (10). Natural and synthetic polymers are used to fabricate the nanofiber structure, creating a three-dimensional environment that could be beneficial for cell attachment and proliferation. However, with the electrospinning technique, small pores are formed, and the porogen technique is not an option for larger structures as porogens need to be totally removed (7).

Three-dimensional printing can also be associated with other techniques, such as freeze-drying, to enhance the mechanical properties and biocompatibility of the scaffold. Kankala and colleagues elaborated a poly(lactide-co-glycolide) (PLGA) scaffold using the extrusion 3D printing technique and immersed it in a gelatin/nano-hydroxyapatite solution (11). The scaffolds were frozen and lyophilized, resulting in a more hydrophilic scaffold with enhanced mechanical properties and biocompatibility, as well as presenting an increased level of alkaline phosphatase activity and higher osteocalcin content. Their study exemplifies an ingenious association of techniques and polymers to achieve a functional scaffold.

### Composition of bone regeneration scaffolds

**Figure 3:**
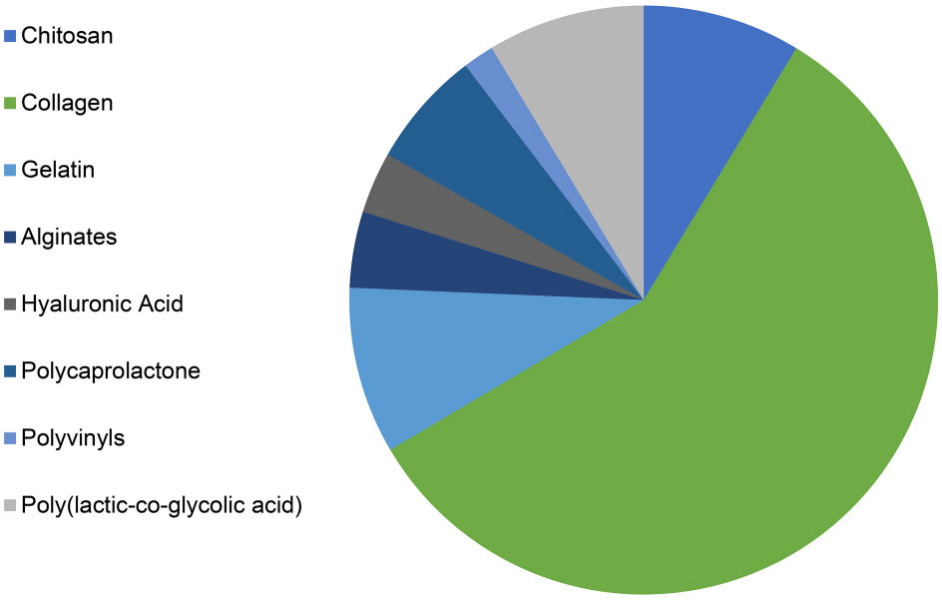
Graph showing polymer distribution in publications retrieved from a search in the PubMed database with the terms: “Chitin”[MeSH], “Collagen”[MeSH], “Gelatin”[MeSH], “Alginates”[MeSH], “Hyaluronic Acid”[MeSH], “Polycaprolactone” [Supplementary Concept], “Polyvinyls”[MeSH], “Polylactic Acid-Polyglycolic Acid Copolymer”[MeSH]. Each term has been associated with AND “Bone Regeneration” [MeSH]. June 1, 2020.

**Figure 4:**
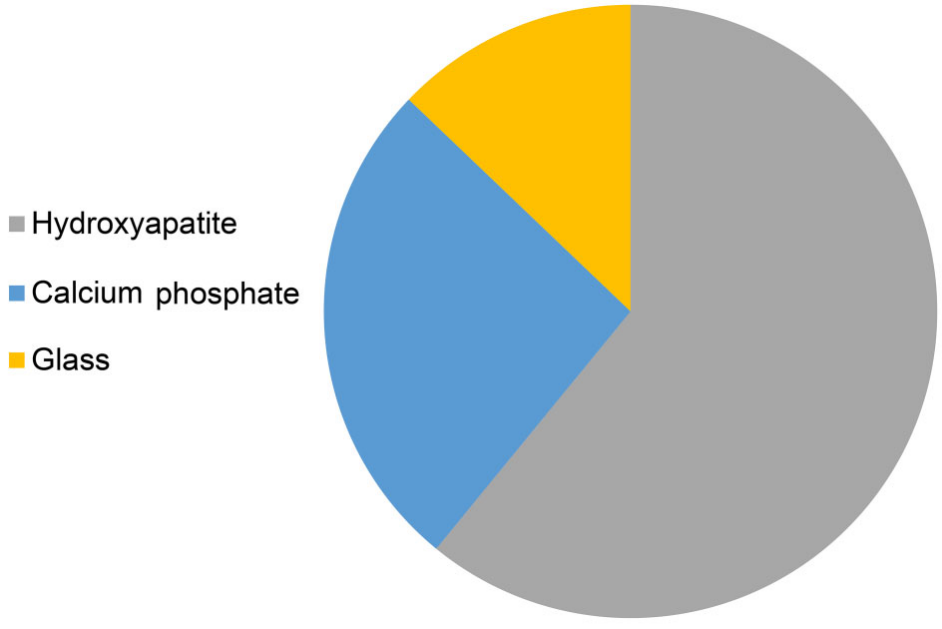
Graph showing ceramic distribution in publications retrieved from a search in the PubMed database using the following terms: “Hydroxyapatites”[MeSH], “Calcium Phosphate”[MeSH], “Glass”]MeSH]. Each term has been associated with AND “Bone Regeneration” [MeSH]. June 1, 2020.

In regard to biomaterials composition, Figure 3 shows that in the last 10 years, collagen was the most cited polymer for the development of bone tissue regeneration scaffolds, followed by gelatin, chitosan, PLGA, polycaprolactone, alginates, hyaluronic acid, and polyvinyls. In the case of ceramics, Figure 4 shows that hydroxyapatite (HA) was the most cited ceramic in studies related to bone regeneration, followed by calcium phosphate (CaP), and glass.

Bioactive ceramics and polymers can be classified as biomaterials due to their compatibility with living tissue. Furthermore, natural and synthetic ceramics are resistant to corrosion and can stimulate new bone formation but are very fragile and have low abrasion resistance. Ceramic biomaterials can include bioactive glasses, HA, calcium silicon, β-tricalcium phosphate (β-TCP), and biphasic calcium phosphate (BCP) (12).

Calcium phosphate-based ceramics are widely used for the fabrication of scaffolds for bone tissue engineering as calcium phosphate is abundant in native human bone. HA is the most frequently used bioceramic in the orthopedic field because of its excellent biocompatibility and osteoconduction properties (13). TCP is also commonly used in this field because it promotes proliferation of osteoprecursor cells. This biomaterial has less stability than HA and shows a faster degradation process and higher solubility (14).

Besides the inorganic matrix, bone is mostly composed of collagen. Therefore, many researchers have used collagen scaffolds for bone regeneration purposes. Collagen can provide a three-dimensional environment, which mimics some bone-forming components and can also increase cell proliferation. It can be associated with other biomaterials, such as bioceramics, carbon, and polymers in order to improve mechanical strength and bone repair (15). Calabrese and collaborators showed in their study that collagen associated with magnesium-enriched HA was beneficial for osteogenic differentiation (16).

Synthetic polymers are also widely utilized to produce scaffolds. In the orthopedics, some of the most common ones are poly ε-caprolactone (PCL), polyglycolic acid (PGA), poly(lactide) (PLA), and PLGA. In addition to their biodegradability, the physicochemical and mechanical properties are comparable to native bone tissue. They can also be easily modified to obtain desired properties for different applications. In the research conducted by Lee and collaborators, PCL and PLGA were associated with TCP and a ceramic material made of sintered and ground duck beak. The study shows that the bone volume percentage of the PCL/PLGA/TCP and PCL/PLGA/duck beak scaffold groups is significantly higher than those of the control groups (17).

In recent years, there has been a growing interest in metal ions for bone scaffold purposes due to their biodegradability and biocompatibility, besides having important roles in tissues physiology. Magnesium (Mg-), iron (Fe), zinc, and their alloys have been the most studied. Wu et al. (18) proposed a three-dimensional composite scaffold with HA and magnesium oxide embedded in fiber of silkworm cocoon and silk fibroin. The addition of 1 wt.% MgO led to the release of magnesium ions, which created a weak alkaline environment that improved the proliferation and osteogenic differentiation of bone marrow stem cells (BMSCs*) in vitro*, as well as promoted *in vivo* bone formation. The authors found that a pH between 7.2-8.5 could promote the proliferation of the BMSCs. On the other hand, zinc incorporation in a synthetic hydrogel-amorphous calcium phosphate composite resulted in a significant increase of extracellular matrix mineralization after 21 days of BMSC culture in a study conducted by Chahal et al. (19).

A summary of the relevant studies in the last ten years is presented in Table 1 (11,17,20–28). To elaborate this table, a search in the PubMed database was performed with the formula: ((“Bone Regeneration”[MeSH]) AND “Tissue Scaffolds”[MeSH]) AND “Orthopedic Procedures”[MeSH]. The search with the 10-year filter resulted in 147 articles (29 reviews).

**Table 1:** Summary of relevant studies involving scaffolds for bone regeneration in the orthopedic field in the last ten years.

Reference, year	Scaffold	Cells	Additive	Manufacturing technique	Study type	Results
(20) 2010	β-TCP	BMSCs	-	-	Femur defects in rabbits (n=64)	Prevascularized tissue-engineered bone grafts led to significantly higher volume of regenerated bone and larger amount of capillary infiltration
(22) 2011	PCL/TCP	BMSCs	BMP-2	Fused deposition	Anterior lumbar interbody fusion - in Yorkshire pigs (n=6)	Solid fusion in PCL/TCP/BMP-2 group comparable to autograft bone
(23) 2013	HA *Sintlife, Engipore*	MSCs	-	Slurry expansion	*In vitro*	Non-stoichiometric MG(2+) and stoichiometric apatites, in granular form, represent a more favorable environment for the growth of cells compared to a non-stoichiometric Mg(2+) apatite, in nanostructured paste.
(24) 2014	Cortical part: Silicon carbide (BioSiC)/Collagen/HA Spongy-like part: Bio-hybrid HA/collagen	BMSCs	PRP	Electrodeposition of collagen, freeze drying	Study in sheep with diaphyseal defects (n=5)	PRP or BMSCs did not further improve the osteotomy healing. Significantly higher values in periosteal callus score in the BioSiC(HaCol)+BMSC group
(25) 2014	β-TCP	Osteoblastic cells	-	Rapid prototyping (RP) 3D	Rabbit radius defects (n=3)	Scaffolds constructed by perfusion seeding and perfusion culture method exhibited better biological properties, significantly higher new bone formation and greater mechanical properties.
(26) 2015	Bioactive glass	-	-	Melting and homemade fiber tower	Study in rat with tibial defect (n=10)	Similar amount of newly formed bone compared with the control group and enhanced expression of RUNX-2 and RANK-L
(17) 2016	PCL/PLGA/TCP, PCL/PLGA/sintered and ground duck beak	-	-	Multi-head deposition system	Study in rabbit with diaphysis defect (n=4)	Bone volume percentage of the PCL/PLGA/TCP and PCL/PLGA/duck beak scaffold groups was significantly higher compared to the control group.
(27) 2017	Pullulan/dextran-based hydrogel and HA/TCP ceramics	BMSCs	-	-	Study in rat with femoral defect (n=10)	The hydrogel showed significant osteogenic properties and rapid resorption.
(21) 2018	Lithium (Li)-nanoHA/ gelatin microsphere (GM)	BMSCs and human umbilical vein endothelial cells (HUVECs)	Erythrogenin (EPO)	Freeze drying	Study in rabbit with femoral head defect (n=15)	The scaffold was able to improve new bone formation, increasing cell proliferation and osteogenesis, and angiogenesis effects.
(28) 2019	Hyperelastic bone (HA and PLGA)	-	-	3D printing and salt-leaching technique	Study in rat with calvaria defect (n=10)	New bone formation surrounding the scaffold struts by 12 weeks.
(11) 2020	Gelatin/nano-hydroxyapatite/ and poly(lactide-co-glycolide)	Osteoblasts (MC3T3-E1)		3D printing and freeze-drying	*In vitro*	Enhanced hydrophilicity, mechanical properties and biocompatibility. Increased level of alkaline phosphatase activity. Higher osteocalcin content. Promotion of the secretion of collagen I.

β-TCP: β-tricalcium phosphate; BMSCs: bone marrow stromal cells; PCL: polycaprolactone; PLGA: poly(lactic-co-glycolic acid); BMP-2: bone morphogenetic protein 2; HA: hydroxyapatite; MSCs: mesenchymal stromal cells; PRP: platelet-rich plasma; RUNX-2: runt-related transcription factor 2; RANK-L: receptor activator of nuclear factor-kappa β ligand; MC3T3-E1: osteoblastic cell line.

### Animal models

Numerous *in vivo* experiments have been conducted in order to prove the effectiveness of the association of scaffolds and stem cells. The analyzed studies in Table 1 demonstrated that rabbits and rats are the most common animals tested in the orthopedic field. In the study carried out by Wang and collaborators (20), rabbits were used as animal models for treating bone defects in the femur. They proposed an association of β-TCP ceramic scaffold and mesenchymal stem cells (MSC). When the femoral vascular bundle was fixed into the side groove of the scaffold seeded with MSC, a higher new bone formation percentage and better vascularization were seen. Li et al. (21 [Bibr B22] [Bibr B23] [Bibr B24] [Bibr B25] [Bibr B26] [Bibr B27]) followed the same strategy with the same animal model but combining the stem cells with different biomaterials and bone morphogenetic proteins (BMP-2), increasing new bone formation. Femoral defects were also conducted in rats, as shown in the study by Johnson and collaborators (29) using polycaprolactone/collagen/heparin scaffolds with the incorporation of BMP-2. Radial bone defect model in rabbits and rat calvaria defect are also frequently used. *In vivo* studies with sheep and pigs are still found although less frequently.

### Clinical trials

Synthetic scaffolds based on HA and βTCP have been the most used in clinical studies in orthopedics. Jäger and collaborators investigated the potency of bone marrow aspiration concentrate (BMAC) to augment bone grafting and support bone healing in local bone defects with a defect area (length × width) measurement larger than 1×1 cm. The BMAC was mixed with porous hydroxyapatite granules (Orthoss^®^) (n=27) or applied onto a collagen sponge (Gelaspon^®^) (n=12). The results show that BMAC-HA has complete bone healing faster than the group of BMAC-collagen and also that the postoperative bone formation appeared earlier in the HA group (30).

In 2016, Šponer et al. (31) reported a prospective, controlled clinical trial (n=9) utilizing expanded autologous MSCs on a ultraporous β tricalcium phosphate synthetic graft material (Vitoss^®^) for femoral defects. The association of the βTCP with MSCs increased the trabecula and decreased the radiolucency within the defect. Later in 2018, Šponer et al. (32) reported a prospective study, this time with 19 patients for the MSCs/βTCP group and using a control group treated with cancellous allografts. No significant difference was observed between femoral defect healing in the MSCs/βTCP group and the cancellous allograft group. However, significant differences were documented between the βTCP group and the cancellous allograft group.

The use of scaffolds in the clinical field has shown good results for bone regeneration purposes; however, translational difficulties still exist and more clinical studies are necessary for the implementation of scaffolds in clinical practice.

## Degradable biomaterials for bone regeneration in dentistry

Bone regeneration is one of the most important and challenging tissue engineering approaches in regenerative medicine, being a promising technique in dentistry as it is considered to be an ideal strategy for treating diseases, injuries, and defects within the maxillofacial region. More importantly, the principles of tissue engineering have been applied in several branches of dentistry, such as oral maxillofacial surgery, periodontics, and implant dentistry, with a wide variety of scaffolds available for purchase on the market.

Tooth extraction caused by periodontal disease or trauma, leading to alveolar bone loss are frequent problems that dentists have to deal with. Bone resorption of the residual ridge continues throughout life in edentulous patients, being difficult to restore the missing teeth with dental implants or prosthodontic approaches (33). In addition, the occurrence of defects in maxillofacial bones are common and can be caused by a range of factors such as infections, congenital deformities, trauma, or tumorectomy (34), which need to be treated for reposition of tissue and restoration of function of the patient. Therefore, tissue engineering techniques using scaffolds for bone regeneration have become a frontier in dentistry, looking towards the preservation of the periodontium and the different bone regions of the maxillofacial complex.

In Table 2, a summary of relevant studies related to scaffolds for bone regeneration in the dentistry field is presented (35-45). A search was performed in the PubMed database with the formula: ((“Bone Regeneration”[MeSH]) AND “Tissue Scaffolds”[MeSH]) AND “Dentistry”[MeSH] on April 16, 2020 using the filter of the last ten years and not considering reviews.

**Table 2:** Summary of relevant studies involving scaffolds for bone regeneration in the dentistry field in the last ten years.

Reference, year	Scaffold	Cells	Additive	Manufacturing technique	Study type	Results
(35) 2010	BMG (bone matrix gelatin) × autogenous bone graft	-	-	Freeze-drying	Study in cats with alveolar osseous defects (n=4).	Greater levels of new formed bone in BMG group. Only on day 56, the mean of bone density was significantly higher in the BMG group.
(36) 2011	β-TCP	-	Platelet-derived growth factor (PDGF)	-	Clinical trial, periodontal osseous defects (n=27).	The linear bone growth and percentage of bone filling were significantly higher in the PDGF+β-TCP group at 6 months compared with that in the β-TCP group.
(37) 2012	β-TCP	-	BMP-7	-	Study in rabbits with osseo-periosteal mandibular defect (n=6).	The overall mean of the percentage of regenerated bone was considerably greater when BMP-7 was incorporated.
(38) 2013	PCL/TCP (80:20) (Osteopore)	BMSC	-	3D printing	Vertical alveolar ridge defect in dogs' mandible (n=4).	Early revascularization and higher amount of new bone.
(39) 2015	β-TCP and Type I collagen	BMSC	-	-	Study in beagle dogs with class III furcation defects (n=6).	BMSC/collagen and BMSC/collagen/β-TCP enhanced periodontal tissue regeneration compared with collagen and β-CP/collagen.
(40) 2015	Magnesium/PLGA	-	-	Solvent casting, salt leaching	Study in beagle dogs with alveolar bone defects (n=6).	Mg provided pH buffering properties to the scaffold, as well as an osteoconductive environment for bone growth.
(41) 2016	Chitosan/β-glycerophosphate with anorganic bovine bone	BMSC	-	Freeze-drying	Study in beagle dogs with bone periodontal defects (n=6).	Highest new bone area value and new bone height value compared with the control group. No significant difference was shown with the incorporation of the cells.
(42) 2017	Fibronectin/decellularized pulp tissue	-	-	Decellularization and freeze-drying	Study in rabbits with calvaria defects (n = 12).	Silk fibroin-coated scaffold demonstrated the ability to induce new bone formation with low inflammation and high vascularity.
(43) 2018	Nano-HA and collagen type I (1:1)(Allgens)+Mg-Ca alloy rods	-	-	Three Mg-Ca alloy rods were insertedinto mineralized collagen	Study in dogs with canine socket preservation model (n=6).	The combined scaffold of mineralized collagen/ Mg-Ca alloy rods was more effective at reducing the absorption of the alveolar ridge and preserving the socket site than the mineralized collagen alone.
(44) 2019	Silica coated nanoHA-gelatin reinforced and poly(L-lactic acid) PLLA	-	-	Electrospun and chemical synthesis route	Study in rabbits with bone defects in the jaw (n=12).	The scaffold suffered degradation along with the regeneration of new tissue.
(45) 2020	PCL/TCP-based ink and methacrylate hyaluronic acid/methacrylate gelatin-based bioink	-	Resveratrol and strontium ranelate	3D printing	Study in rats with critical-sized mandibular bone defect (n=6).	Enhanced angiogenesis and inhibition of osteoclast activity. The scaffolds promoted MSC osteogenic differentiation and bone formation

BMG: bone matrix gelatin; β-TCP: β-tricalcium phosphate; TCP: tricalcium phosphate PDGF: platelet-derived growth factor; BMSCs: bone marrow stromal cells; PCL: polycaprolactone; PLGA: poly(lactic-co-glycolic acid); PLLA: poly(L-lactic acid); BMP-7: bone morphogenetic protein 7; HA: hydroxyapatite; Mg: magnesium; Ca: calcium.

As shown in Table 2, several biomaterials have been proposed for maxillofacial bone regeneration including synthetic polymers such as PCL, PLLA, and PLGA, calcium phosphate compounds such as βTCP, biphasic calcium phosphate (HA/β-TCP), cyanoacrylate-combined calcium phosphate (CCP), and HA and its compounds such as MgHA or n-HA/PA. Natural polymers have also been proposed such as collagen, hyaluronic acid, PRF, ilk fibroin, gelatin, and chitosan, and even decellularized matrices such as cartilage matrix or demineralized bone matrix. However, only HA, TCP, inorganic bone matrix, and collagen scaffolds have been widely evaluated clinically as bone substitutes to date (33,46).

HA and TCP ceramics have a chemical and biological similarity to bone tissue, providing good biocompatibility and cell adhesion properties. TCP is a resorbable biomaterial with a faster degradation rate than bone regeneration (47). It has therefore been necessary to improve its mechanical properties by the association with other biomaterials such as HA or PCL (38,39,48). β-TCP releases calcium ions into local tissue, which contributes to the control of osteoblastic viability, proliferation, and differentiation (49), indicating that calcium phosphate-based materials present osteoconductivity properties.

A human clinical study with X-ray computed microtomography (microCT) and histomorphometric analysis of bone biopsies demonstrated that for sinus augmentation, 9 months after grafting a scaffold of HA/β-TCP 30/70 in granules or blocks, the block-based scaffold had a significantly higher strut thickness and strut number, closer to that of native healthy tissue (48). In addition, a large amount of newly formed bone and a rich net of new vessels was detected, which meant that not only the scaffold composition but also the scaffold morphology influences the quality of the regenerated tissue.

Although the sole implantation of scaffolds has brought good results, studies have shown that planting stem cells on scaffolds, previous to the implantation, contributes to bone deposition and angiogenic stimulation, which could be relevant in poor bone marrow sites such as the mandibular angle (38,39,41,50). Additionally, in cases where dental implants are placed in junction with bone grafts, bone-implant contact has also improved with the addition of stem cells on the scaffold, contributing to primary stability of the dental implant (49,51).

Growth factors like GDF-5, BMP-7, PDGF, and BMP-2 have also been incorporated onto scaffolds, improving therapeutic potential and increasing the percentage of the regenerated bone area (36,37,52).

Despite the latest technological advances achieved in bone tissue scaffolds, autogenous bone is still the gold standard in dentistry, but limitations related to bioavailability and morbidity makes continuous research necessary in order to develop a scaffold resembling the autogenous bone matrix. However, Bayat and collaborators used BMG to regenerate alveolar osseous defects in cats, showing new bone formation and mineralization superior to autogenous bone at day 56 (35). Additionally, the use of calcium phosphate cement with the addition of cryopreserved bone-derived osteoblasts has shown results comparable to that of autogenous bone.

As in orthopedisc, three-dimensional printing in dentistry has assumed important relevance. It has many advantages such as the possibility of direct printing in the defective site, which means a faster scaffold preparation with increased accuracy. It also allows the production of scaffolds with different intricate shapes resembling the lesion.

Recently, Lopez and collaborators developed a three-dimensionally printed bioactive ceramic scaffold of β-TCP coated with dipyridamole, an adenosine A2A receptor indirect agonist, which could inhibit osteoclastogenesis. They created a critical-size bone defect at the mandibular rami of rabbits. Bone growth was evaluated by microCT. The results showed a larger percentage of bone in the dipyridamole group, which means that incorporation of dipyridamole increased the bioactivity of the scaffold (53).

## Commercially available scaffolds

The collagen membranes type I or III from porcine or bovine origin have been widely used for guided bone regeneration, which consists of creating a barrier to prevent the migration of soft tissue cells into the bone graft. Without the presence of such a barrier, the cells could invade the environment for the new bone formation (50).

Analyzing the studies obtained after applying the same formula cited above ((“Bone Regeneration”[MeSH]) AND “Tissue Scaffolds”[MeSH]) AND “Dentistry”[MeSH] on April 4, 2020) on the PubMed database, it was found that the composition of the majority of the scaffolds used in the studies was the natural polymer collagen, and commercially available scaffolds such as Olympus Terumo Biomaterials, Parasorb cone, Terudermis, Bio-Gide, CollaCote, BD™ 3D Collagen Composite Scaffold, and Mucograft were used. Also, tilapia type I collagen scaffold has been studied showing promising results (54). Additionally, hybrid bone substitutes based on hydroxyapatite/TCP were reported as Osteon II, Ceraform^®^, and Reprobone.

Mineralized collagen has been studied due to its similarity to the natural bone microstructure because it is composed of nano-hydroxyapatite and collagen type I (1:1). To compensate mechanical limitations, three Mg-Ca alloy rods were inserted into mineralized collagen, a strategy that allows for a successful overcoming of such a limitation (43).

For vertical fillings or socket filling, deproteinized bovine bone is the scaffold most frequently used due to its osteoconduction properties and bone neoformation capacity. Although it is not fully reabsorbed by the body, clinical success in regard to good structural support has been reported in the scientific literature (55). Bio-Oss is an example of this type of scaffold, being the commercial scaffold of choice for many bone regeneration procedures in dentistry. However, Mayer and collaborators demonstrated that a bioactive bovine bone (Alpha Bio's Graft) scaffold reinforced with PCL caused a higher percentage of new bone compared to Bio-Oss (56).

## Animal models

Several animal model studies have been used to evaluate scaffolds in which cats, rabbits, rats, and dogs were used to develop various types of bone defects (Table 2). Bone defects frequently used in the dentistry field are alveolar osseous defects, peri-implant defects, periodontal osseous defects, dehiscence type defects in mandible, bone alveolar ridge defects, class III furcation defects, calvaria defects, maxillary sinus floor augmentation, and mandibular branch defects, which are the most frequent lesions that dentists have to deal with.

Scaffolds (BMG or magnesium/PLGA) without cells or growth factors successfully promoted new bone formation in models of alveolar bone defects in cats and dogs (35,40) and also in models of peri-implantitis defects in rabbits (scaffold silk fibroin powder/PRF) (57). Furthermore, in a study using a periodontal defect model in dogs and a chitosan/β-glycerophosphate scaffold, the incorporation of BMSC did not represent a significant increase in new bone area or new bone height (41), showing a similar performance compared with the group without cells.

## Clinical trials

Clinical studies with humans have demonstrated that for bone regeneration of small bone defects, such as alveolar bone defects (58) or some sinus augmentations (48), the scaffold implantation is sufficient, without the need of cells or growth factors. However, the use of cells and growth factors could accelerate healing, as it is interesting to reduce the waiting time between the surgical-prosthetic steps (59). Rickert and collaborators showed that for maxillary sinus floor elevation, the incorporation of stem cells into a bovine bone mineral scaffold (BioOss^®^) was a strategy as efficient as using BioOss^®^ mixed with autogenous bone (46). Nevertheless, Chen et al. (60) showed that for periodontal defects, no statistically significant difference was detected when stem cells are incorporated into the BioOss^®^ scaffold in a randomized clinical trial.

Jayakumar et al. (36) carried out a human clinical study, where patients presented periodontal osseous defects. Their strategy to gain bone volume was to incorporate PDGF into a scaffold of β-TCP. The results showed that linear bone growth and percent of bone filling were significantly higher in the PDGF-BB+β-TCP group at the end of 6 months, compared with that in the β-TCP group.

Since there is a range of possibilities of techniques and materials for the production of bone regeneration scaffolds, further studies are suggested with a higher number of patients and applying a randomization criterion. In addition, it is necessary to establish which type of cells and growth factors are more appropriate to be associated with scaffolds and also to establish the most appropriate technique for scaffold production according to the bone morphology and localization.

## Role of metallic biomaterials in orthopedics and dentistry

Metallic biomaterials are widely used in orthopedics as support devices, being mainly utilized for the manufacture of plates, fixation screws, and orthopedic implants for the replacement of missing joints or bones. In the dentistry field, metallic biomaterials can be also used to manufacture dental implants as well as metallic meshes for the stabilization of bone grafts in guided bone regeneration. Its rigidity provides space maintenance and avoids collapse of the contour and displacement of the graft.

The success of a metallic implant in the human body is directly related to biocompatibility and reduced immune response. Despite the widespread use of metal implants, they have some disadvantages and although being biotolerable or bioinert, metal alloys can release particles due to wear or chemical degradation, which could cause different pathologies, requiring a posterior removal of the implant (61), and even stimulate the metabolic pathways of many cell types, including osteoclasts, osteoblasts, lymphocytes, macrophages, or fibroblasts (62). In addition, the failure of implants can occur due to the difference between the elastic modules of the metallic implants and the host tissue, causing the phenomenon known as “stress shielding” (63).

**Figure 5:**
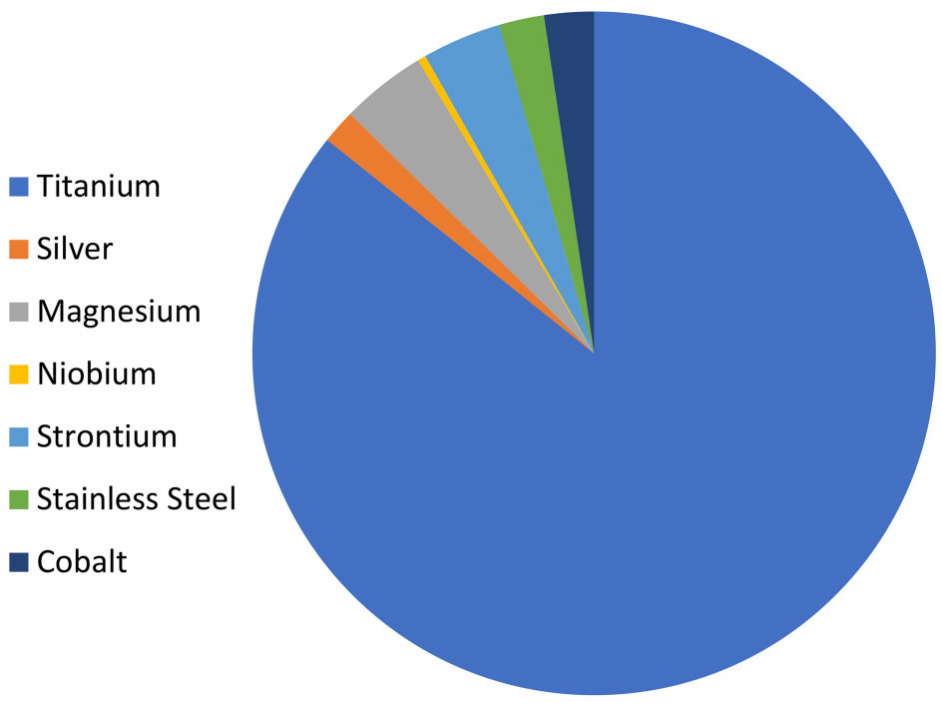
Graph showing metals distribution in publications retrieved from a search in the PubMed database using the following terms: “Titanium”, “Silver”, “Magnesium”, “Niobium”, “Strontium”, “Stainless Steel” and “Cobalt”. Each term has been associated with “AND “Bone Regeneration” [MeSH]. June 1, 2020.

Among the metallic biomaterials, the use of silver, magnesium, cobalt, niobium, strontium, and titanium is becoming prominent for application in bone regeneration in dentistry and orthopedics. However, for support elements, titanium is the most commonly used (64) (Figure 5); among the titanium alloys, Ti6Al4V is the best known, being used in approximately 50% of the applications (65).

Titanium and its alloys are called “special metals” and are considered ideal for implants as they have satisfactory mechanical properties and resistance to corrosion. The latter property is normally conferred to these materials due to the occurrence of a spontaneous reaction with the formation of a passive layer of TiO_2_ on its surface, which acts as a protective barrier.

However, depending on the conditions of the medium, titanium and its alloys can corrode very quickly or slowly depending on the environment being anaerobic, where water is the oxidizing agent, or aerobic, where oxygen is the oxidizing agent. In the case of alloys, the thickness of this oxide layer becomes a critical parameter because it acts directly on controlling the release of metal ions (66,67). If this layer is very thin, the release of these ions is gradually facilitated, and thus it becomes less protective. On the other hand, the thicker the layer is, the more likely is crack formation, which will eventually lead to a pronounced release (66). In extreme cases, corrosion can cause a reduction in the mechanical performance, or even fracture of the implant, reaching the surrounding tissue, and consequently, the need for implant removal (67).

In the specific case of Ti6Al4V, some *in vivo* studies have already shown the release of titanium ions by the dissolution of implanted devices (68). The dissolution of aluminum (Al) in the human organism can promote pathologies such as the Alzheimer's disease, peripheral neuropathy, and osteomalacia. In addition, the presence of vanadium (V) can alter the kinetics of enzyme activity associated with the inflammatory response (69).

Another important factor in relation to titanium implants and their alloys is that their surfaces constitute an adequate environment for bacterial adhesion and proliferation, favoring the formation of biofilms that are largely inaccessible to the immune system and resistant to the action of drugs (70). Biofilm is a biological system layer that naturally develops from the adhesion of microorganisms to the surface; these biofilms are related to certain infectious diseases, and consequently to implant failure (71).

Qiao et al. (72) proposed a three-dimensional printed porous titanium alloy (Ti_6_Al_4_V) embedded in an antibacterial hydrogel to prevent infections. The porous structure made the implant more compatible with host bone tissue, as it avoided stress-shielding and osteolysis. Furthermore, the implant system demonstrated effective antibacterial properties while inducing bone repair and osseointegration. This study shows that a non-degradable biomaterial can be associated with a degradable one to enhance tissue repair.

Therefore, the superficial modification of metallic implants is essential for improving their electrochemical performance, biocompatibility, and osseointegration process, with the aim of increasing their long-term stability as a biomaterial (73).

## Surface treatments

Several processes to alter the surface characteristics of metallic implants in order to improve adaptation to bone tissue after implantation have been used.

The surface of metals can be modified with inorganic coatings such as CaP. These kinds of coatings allow for enhanced corrosion resistance, reduced metal ion release, and osteoblast attachment promotion. Oliveira and colleagues evaluated a nano-hydroxyapatite coated implant in diabetic rats, showing a statistically significant difference in gene expression of osteogenic markers Runx2, alkaline phosphatase, osteopontin, and osteocalcin in the early stage of osseointegration (74). Mokabber and colleagues developed a silver/calcium phosphate coating via electrochemical deposition on titanium substrates. The biomaterial showed bacterial reduction and excellent compatibility when silver ions were deposited as metallic silver nanoparticles on the CaP coating (75). Besides the CaP-based coatings, other inorganic coatings have been proposed such as glass ceramics, zeolites, and carbon, which possess great potential for bone tissue engineering.

Composite coatings have also been proposed to obtain enhanced bone regeneration properties, and they can usually be combined with inorganic coatings. Yu et al. (76) coated Ti-Al-4V substrates with collagen-HA composites, resulting in a slightly higher osteoblast proliferation rate compared with HA coating.

Organic materials such as synthetic and natural polymers can be used as coatings for metallic implants. Polymers can prevent the corrosion of the implant, in addition to improving cell viability and adhesion. Also, polymer coatings could be used for the release of drugs that could contribute to the osseointegration of the implant. Therefore, anti-inflammatories have been proposed to prevent the aseptic loosening of orthopedic implants (77). Aseptic loosening is a frequent problem that occurs when particles are worn away from the implant surface and stimulate aseptic inflammatory responses for the phagocytosis of those small wear particles. Moreover, polymer coatings for the release of drugs that could support bone formation, such as alendronate and albumin, have been proposed (78).

On the other hand, the electrochemical treatments of electropolishing and anodizing, coatings obtained by plasma electrolytic oxidation, silane hybrid coatings by sol-gel, and plasma polymerization are prominent for application in the area of tissue regeneration.

In the electropolishing process, an anodic leveling and anodic brightening of the surface is made to reduce the roughness and improve the resistance to corrosion (79). This is considered an electrochemical process carried out through the use of the metallic substrate as a working electrode for polishing the metal (80). This process is performed using a voltage source that produces an electric current that passes from the anode to the cathode, resulting in the oxidation of the metal and removal of material from the surface at a controlled rate (81). The amount of the removed metal depends on the electrolyte, temperature, current density, and the metal to be electropolished (80).

The anodizing process aims to produce a stable oxide layer that is firmly adhered to the metal substrate. On titanium surfaces and their alloys, the formation of this layer normally occurs with a nanometric and self-organized topography as nanotubes, which has shown to be a promising strategy for biological and osseointegration processes (82). The main electrochemical parameters that affect the formation of these porous structures are pH, voltage, temperature, and the presence of impurities in the material. The anodizing voltage itself mainly controls the diameter of the pore or tube. At the beginning of the electrochemical oxidation, the entire surface of the metal is covered with a compact and uniform anodic oxide, and following this, the layer begins to adsorb anions from the solution, promoting the formation and growth of the nanotubes (83).

The surface modification by plasma-assisted anodizing (PEO) treatment has been shown to be an innovative technology for the biomedical field (84). It is considered to be an electrochemical conversion treatment, which provides the superficial formation of a metallic oxide layer. It is a technique analogous to anodizing, but using higher values of current potentials and current densities, which causes a plasma formation on the sample surface. These coatings offer some advantages such as the adjustment of the elements incorporated into the metallic matrix and the control of the microstructure according to thickness and porosity (85).

Hybrid silane-based coatings combine organic and inorganic silicon-based functional groups, whose general formula is R'(CH2) nSi (OR) 3, where R' is an organofunctional group and R is a hydrolyzable alkoxy group. When in contact with water, silanes are hydrolyzed to form silanol groups (SiOH), which allow the bonding to the hydrated metal surface (metal-OH) via the formation of Si-O-metal bonds. The silanol groups undergo self-crosslinking through siloxane (Si-O-Si) bonds, resulting in an organic protection layer chemically bonded to the metallic substrate (86). Specifically, many of these coatings are being studied to improve the corrosion performance of metallic prostheses or to functionalize their surfaces through the incorporation of bioactive particles, biomolecules, drugs, and/or organic components dispersed in silane precursors (70,87).

Another important application of silane coating in the biomedical field is the release of silicon compounds by hydrolytic degradation of the sol-gel network. The presence of the silicon element assists in the connective tissue metabolism of bone and cartilage and is associated with bone formation and calcification. In addition, silicon is involved in collagen type I synthesis and osteoblastic differentiation (88).

An advanced sol-gel technique is the application of hybrid silane coatings by the plasma polymerization technique, creating a film that protects the implant from corrosion, as it can be highly cross-linked and insoluble. The plasma can be used to chemically decompose the silane precursor and as a source of an active species to promote the formation of the film composition close to the used monomer (precursor) (89). Important advantages of this method are that it does not need toxic solvents, precursors do not necessarily need to present unsaturation to propagate the polymerization, and the adjustment of the parameters used in the plasma allows for the control of the properties of the resulting materials (thickness and chemical composition) (90). It is noteworthy that the use of alkoxysilane precursors stands out in this application method, mainly due to vaporization at room temperature, but some studies have been prioritizing the use of organoalkoxysilanes (89). In addition, thanks to easy thickness control, nanoscale coatings can be obtained, which consequently can favor protein interactions with bone cells and cell differentiation.

## Final considerations

Despite the great advances in bone tissue engineering, the translation of innovative bone healing strategies to clinical applications still has a long way to go. The incorporation of cells has shown interesting results for bone tissue regeneration, but cell therapies are difficult to translate due to the complex regulatory barriers. Furthermore, the preparation of cells before implantation represents additional work, which is still not cost effective.

Currently, a large majority of studies have performed *in vivo* tests with smaller animals such as rats and rabbits, but testing these biomaterials in large animal models will be very important to analyze their effects more appropriately.

Furthermore, in the field of dentistry, in which irregular-shaped critical-size defects are common in the craniofacial region, the 3D printing technique will be relevant, but the reduction of production costs will be necessary for clinical implementation.

Another relevant point to consider is that the microbial load in the oral cavity makes the development of scaffolds with antimicrobial function for the control of the infection and the promotion of bone formation critical.

Finally, since appropriate vascularization is indispensable for osteogenic differentiation, it is important to continue working on scaffolds that allow for vascularization in order to obtain a fully mature bone.

With regard to metallic biomaterials, although there are various surface modification techniques, many are costly and difficult to execute, which limit their clinical translation. Thus, it is important to look for simple and cost-effective strategies to achieve osteoconductive surfaces that are also capable of inhibiting the formation of biofilms.
